# Death Cafes: Brewing Up Conversations about Death

**DOI:** 10.1093/geroni/igaf122.2607

**Published:** 2025-12-31

**Authors:** Abby Schellhorn, Melinda Heinz

**Affiliations:** University of Northern Iowa, Elkader, Iowa, United States; University of Northern Iowa, Cedar Falls, Iowa, United States

## Abstract

Death can be a difficult topic to talk about and conversations about the idea seem taboo. Death Cafes began in the U.K. in 2011 and in the U.S. in 2016. Since 2023, 13 Death Cafes in a university town in the Midwest were offered (N = 101). Survey results revealed participants were between the ages 19 to 85, including community members, college students, and professors. Approximately 69 percent were women, 22 percent were men, 2 percent were transgender, and 7 percent were gender non-conforming. Participants reported a range of religious and spiritual beliefs. Approximately 48 percent indicated that they were not religious, but 82 percent were spiritual; 51 percent were religious, and 16 percent were not spiritual. When participants were asked about why they attended, a range of responses were given including: “Invited by a friend, sounded interesting, curiosity” and “to learn more about preparing for death.” As attendees reflected, participants mentioned they enjoyed the open conversation without fear of being shut down for talking about death. One participant said, “It was great! People seemed free to discuss anything death related and everyone seemed open to discussion without judgment.” Many participants also commented that they “hoped to attend more in the future.” Death Cafes allow attendees the freedom to talk about difficult topics in a respectful environment where diverse viewpoints and perspectives are welcomed. These findings have implications for gerontologists and show the importance of creating safe and welcoming spaces for adults of different ages to discuss death.

